# Measurement-based evaluation of Google/Apple Exposure Notification API for proximity detection in a light-rail tram

**DOI:** 10.1371/journal.pone.0239943

**Published:** 2020-09-30

**Authors:** Douglas J. Leith, Stephen Farrell

**Affiliations:** School of Computer Science & Statistics, Trinity College Dublin, Dublin, Ireland; University of Pisa, ITALY

## Abstract

We report on the results of a Covid-19 contact tracing app measurement study carried out on a standard design of European commuter tram. Our measurements indicate that in the tram there is little correlation between Bluetooth received signal strength and distance between handsets. We applied the detection rules used by the Italian, Swiss and German apps to our measurement data and also characterised the impact on performance of changes in the parameters used in these detection rules. We find that the Swiss and German detection rules trigger no exposure notifications on our data, while the Italian detection rule generates a true positive rate of 50% and a false positive rate of 50%. Our analysis indicates that the performance of such detection rules is similar to that of triggering notifications by randomly selecting from the participants in our experiments, regardless of proximity.

## 1 Introduction

There is currently a great deal of interest in the use of mobile apps to facilitate Covid-19 contact tracing, see e.g. [1–3]. The basic idea of a contact tracing app is that if two people carrying mobile handsets installed with the app spend significant time in close proximity to one another (e.g. spending 15 minutes within 2 metres) then the apps on their handsets will both record this contact event.

Contact tracing apps based on the Google/Apple Exposure Notification (GAEN) API [4] are currently being rolled out across Europe, with apps already deployed in Italy, Switzerland and Germany. These apps use Bluetooth received signal strength to estimate proximity and will likely be used as an adjunct to existing manual contact tracing and test systems. Existing manual systems can usually readily identify the people with whom an infected person share accommodation and with work colleagues with whom the infected person is in regular contact. More difficult is to identify people travelling on public transport with whom an infected person has been in contact, since the identities of these people are usually not known to the infected person and are generally not otherwise recorded. Public transport is therefore potentially an important use case where effective contact tracing apps may be of significant assistance in infection control.

We report on the results of a Covid-19 contact tracing app measurement study carried out on a commuter tram. The tram is of a standard design widely used in Europe. Measurements were collected between 108 pairs of handset locations and are publicly available [5].

In summary, our measurements indicate that in the tram there is little correlation between received signal strength and distance between handsets. Similar ranges of signal strength are observed both between handsets which are less than 2m apart and handsets which are greater than 2m apart (including when handsets are up to 5m apart). This is likely due to reflections from the metal walls, floor and ceiling within the tram, metal being known to be a strong reflector of radio signals [6, 7], and is coherent with the behaviour observed on a commuter bus [8].

We applied the detection rules used by the Italian, Swiss and German contact tracing apps to our measurement data and also characterised the impact on performance of changes in the parameters used in these detection rules. We find that the Swiss and German detection rules trigger no exposure notifications, despite around half of the pairs of handsets in our data being less than 2m apart. The Italian detection rule has a true positive rate (i.e. correct detections of handsets less than 2m apart) of around 50%. However, it also has a false positive rate of around 50% i.e. it incorrectly triggers exposure notifications for around 50% of the handsets which are greater than 2m apart. This performance is similar to that of triggering notifications by randomly selecting from the participants in our experiments, regardless of proximity.

We observe that changing the people holding a pair of handsets, with the location of the handsets otherwise remaining unchanged, can cause variations of ±10dB in the attenuation level reported by the GAEN API. This is pertinent because this level of “noise” is large enough to potrentially have a substantial impact on proximity detection.

## 2 Methodology

### 2.1 Ethical approval

The experimental protocol was reviewed and approved by the Ethics Committee of the School of Computer Science and Statistics, Trinity College Dublin. The ethics application reference number is 20200503. Oral consent was obtained from participants.

### 2.2 Experimental protocol

**Fig 1:**
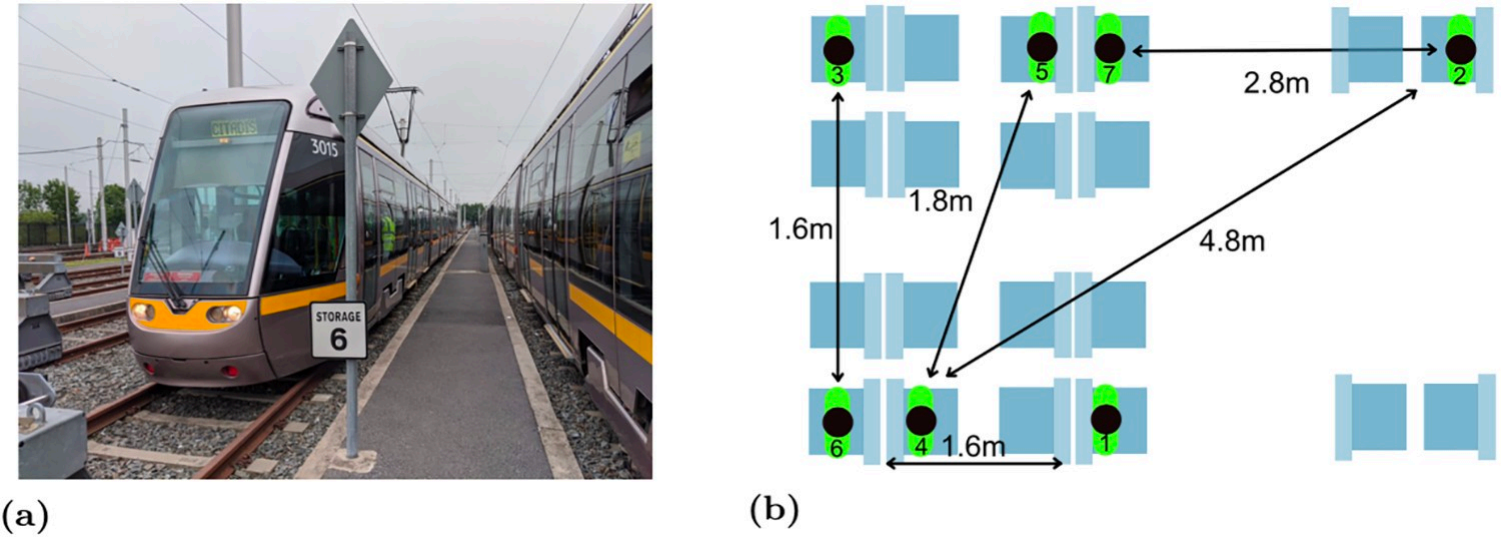
(a) Tram on which measurements were collected. (b) Relative positions of participants during tests.

**Fig 2:**
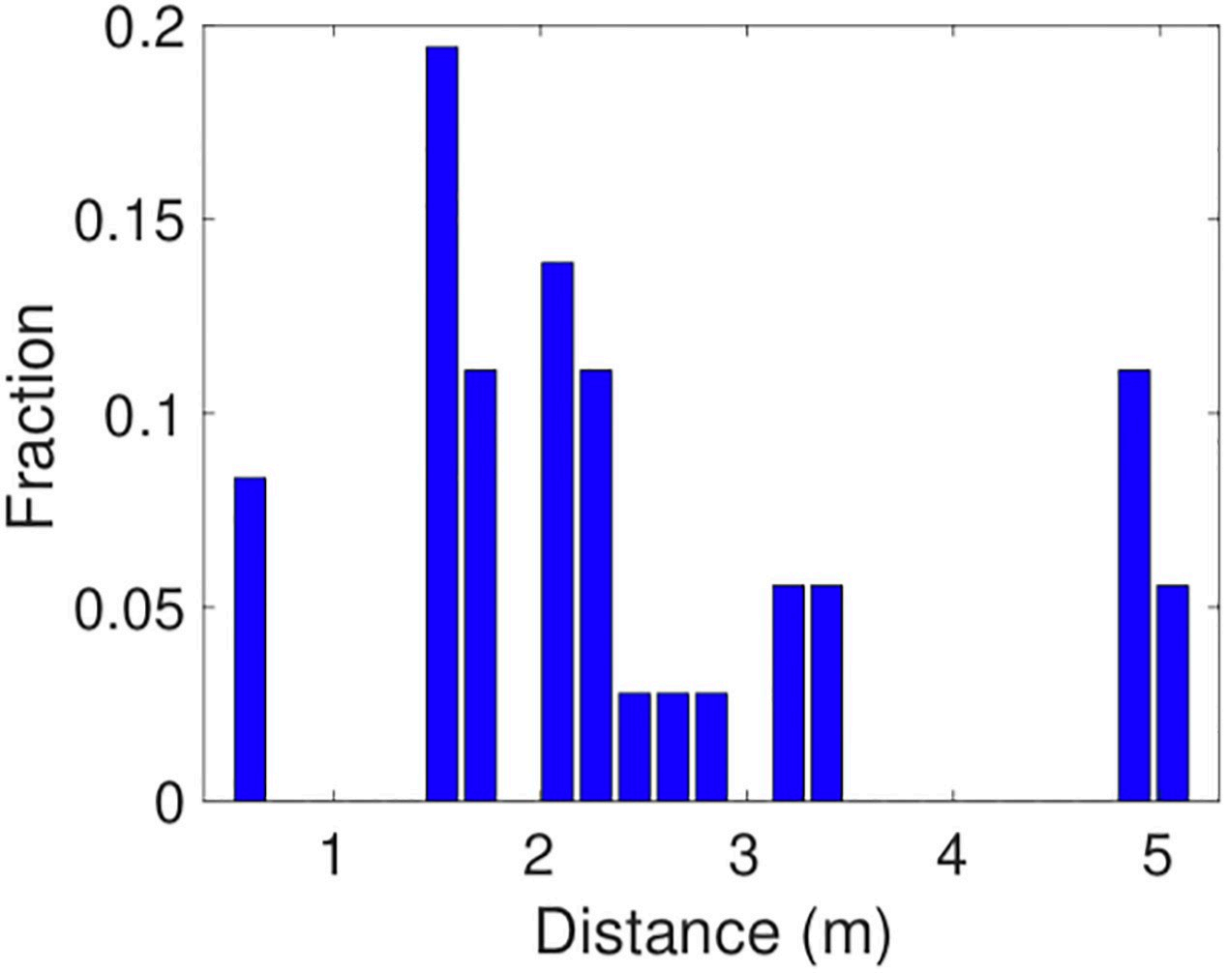
Distribution of distances between participants in experiments.

Our experimental measurements were collected on a standard light-rail tram carriage used to carry commuters in Dublin, Ireland, see Fig 1(a). We recruited seven participants and gave each of them Google Pixel 2 handsets. We asked them to sit in the relative positions shown in Fig 1(b). This positioning aims to mimic passengers respecting the relaxed social distancing rules likely during easing of lockdown and with the distances between participants including a range of values < 2m and a range of values > 2m, see Fig 2. Each experiment is 15 minutes duration giving around 3 scans by the GAEN API when scans are made every 4 mins (per measurements reported in [9]). A Wifi hotspot was set up on the tram and the participants were asked to hold the handset in their hand and use it for normal commuter activities such as browsing the internet.

After the first experiment was carried out participants were then asked to switch seats (they chose seats themselves) and a second 15 minute experiment run. After the second experiment participants were again asked to change seats for the third 15 minute experiment and, in addition, two participants were asked to place their handsets in their left trouser pocket (in an orientation of their choice).

Each handset had the GAEN API and a modified version of the Google exemplar Exposure Notification app [10] installed, and was registered to a gmail user included on the Google GAEN whitelist so as to allow use of the GAEN API by the Exposure Notification app. Each handset also had a GAENAdvertiser app developed by the authors installed. This app implements the transmitter side of the GAEN API and allowed us to control the TEK used and also to start/stop the broadcasting of Bluetooth LE beacons.

At the start of each 15 minute experiment participants were asked to configure the GAENAdvertiser app with a new TEK and then to instruct the app to start broadcasting GAEN beacons. At the end of the experiment the GAENAdvertiser stopped broadcasting beacons. In this way a unique TEK is associated with each handset in each experiment, and these can be used to query GAEN API to obtain separate exposure information reports for each handset in each experiment.

Following all three experiments the handsets were collected, the TEKs used by each handset extracted and the GAEN API on each then queried for exposure information relating to the TEKs of the other handsets. In total, therefore, from these experiments we collected GAEN API reports on Bluetooth LE beacon transmissions between 108 pairs of handset locations. This measurement data is publicly available [5].

To provide baseline data on the radio propagation environment we also used the standard Android Bluetooth LE scanner API to collect measurements of RSSI as the distance was varied between two Google Pixel 2 handsets placed at a height of approximately 0.5m (about the same height as the tram seating) in the centre aisle of the tram carriage.

### 2.3 Hardware & software used

We used Google Pixel 2 handsets running GAEN API version 202512001 As reported in the *Settings-COVID 19 Notifications* handset display, which includes a major update by Google issued on 13th June 2020.

We used a version of the Google exemplar Exposure Notification app modified to allow us to query the GAEN API over USB using a python script (the source code for the modified app is available on github [10]).

In addition we also wrote our own GAENAdvertiser app that implements the Bluetooth LE transmitter side of the GAEN API [4]. GAENAdvertiser allows us to control the TEK, and in particular reset it to a new value at the start of each experiment. In effect, resetting the TEK makes the handset appear as a new device from the point of view of the GAEN API, and so this allows us to easily collect clean data (the GAEN API otherwise only resets the TEK on a handset once per day). We carried out extensive tests running GAENAdvertiser and the GAEN API on the same device to confirm that under a wide range of conditions the responses of the GAEN API on a second receiver handset were the same for beacons from GAENAdvertiser and the GAEN API, see [9] for further details. Subsequent to our measurement study Google has now published the code for the transmitter side implementation and details of the receiver side attenuation calculation [11, 12]. These also confirm that the GAENAdvertiser implementation is essentially identical to the Google transmitter-side implementation.

GAENAdvertiser is open source and can be obtained by contacting the authors (we have not made it publicly available, however, since it can be used to facilitate a known replay attack against the GAEN API [13]).

### 2.4 GAEN use of Bluetooth for proximity detection

The basic idea of a contact tracing app is that if two people carrying mobile handsets installed with the app spend significant time in close proximity to one another (e.g. spending 15 minutes within 2 metres) then the apps on their handsets will both record this contact event. If, subsequently, one of these people is diagnosed with Covid-19 then the contact events logged on that person’s handset in the recent past, e.g. over the last two weeks, are used to identify people who have been in close contact with the infected person. These people might then be made aware of the contact and advised to self-isolate or take other appropriate precautions. For this approach to be effective it is, of course, necessary that the app can accurately detect contact events.

The GAEN API uses Bluetooth LE wireless technology as the means for detecting contact events. Bluetooth LE devices can be configured to transmit *beacons* at regular intervals. To distinguish between beacons sent by different handsets each handset running GAEN generates a random *Temporary Exposure Key* (TEK) once a day. This TEK is then used to generate a sequence of *Rolling Proximity Identifiers* (RPIs), approximately one for each 10 minute interval during the day (so around 144 RPIs are generated). The GAEN system running on a handset transmits beacons roughly every 250ms. Each beacon contains the current RPI value. Approximately every 10 minutes the beacons are updated to transmit the next RPI value. By constantly changing the content of beacons in this way the privacy of the system is improved. In addition to the RPI each beacon also carries encrypted *metadata* containing the wireless transmit power level used. Although beacons are emitted roughly every 250ms, on the receiving side, devices only scan for beacons roughly every 4 minutes [9].

The basic idea is that the signal strength with which a beacon is received provides a rough measure of the distance between transmitter and receiver. Namely, when the received signal strength is sufficiently high then this may indicate a contact event and, conversely, when the received signal strength is sufficiently low then this may indicate that the handsets are not in close proximity. This is based on the fact that in general the radio signal gets weaker as it travels further since the transmit power is spread over a greater area. However, many complex effects can be superimposed upon this basic behaviour. In particular, obstacles lying on the path between the transmitter and receiver (furniture, walls etc) can absorb and/or reflect the radio signal and cause it to be received with higher or lower signal strength. A person’s body also absorbs radio signals in the 2.4 GHz band used by Bluetooth LE and so the received signal strength can be substantially reduced if their body lies on the path between the transmitter and receiver. The relative orientation of two handsets can strongly affect the received signal strength owing to way antennae are packaged within the handset body. In indoor environments walls, floors and ceilings can reflect radio signals even when they are not on the direct path between transmitter and receiver, and so increase or decrease the received signal strength. See, for example, [14] for measurements illustrating such effects in real environments.

### 2.5 Querying the GAEN API

The GAEN system presents an interface to health authority apps This interface allows these apps to submit a request that includes an Exposure Configuration data structure to the GAEN system [4]. The Exposure Configuration data structure allows specification of the TEK to be queried, the start time and duration of the interval of interest (specified in 10 minute intervals since 1st Jan 1970) and a low and high attenuation threshold (specified in dB). The GAEN system responds with one or more Exposure Information data structures that report an exposure duration (field durationMinutes) and an array with three *atttenuation duration* values, giving the duration (in minutes) that the attenuation level is below the low threshold, the duration the attenuation level is between the low and high thresholds and the duration above the high threshold. It is also possible to query for an Exposure Summary response, but we did not make use of this since the relevant information that this contains can be derived from the Exposure Information reports.

For each TEK and time interval we made repeated queries to the GAEN API holding the low threshold constant at 48dB and varying the high threshold from 49dB to 100dB (in 1dB steps up to 80dB, then in 5dB steps since noise tends to be higher at higher attenuation levels). By differencing this sequence of reports we can infer the attenuation duration at each individual attenuation level from 48dB through to 100dB.

At the time of our meaurement study the GAEN documentation did not precisely state how the attenuation level is calculated, nor did it give details as to how the attenuation duration is calculated. The analysis in [9], indicated the attenuation level is calculated as *P*_*TX*_ − *P*_*RX*_, where *P*_*TX*_ is the transmit power level sent in the beacon metadata and *P*_*RX*_ is given by a filtered RSSI For Google Pixel 2 handsets (and others) the RSSI is recorded only from beacons transmitted on one of the three radio channels used by Bluetooth LE for transmitting beacons, see [9]. measurements plus a calibration offset. Google has subsequently published documentation [12] that confirms this.

For the Google Pixel 2 handsets and GAEN API version 202512001 used in our experiments *P*_*TX*_ is -31dB and the calibration offset is -6dB. Google supplied us with the calibration and offset values used for all handset models in GAEN version 202512001 and we have posted these in our online study archive [5]. Note that we observed that the noise floor (the RSSI below which beacons can no longer be reliably decoded) is around -100dB in a Pixel 2, giving a maximum measureable attenuation of around 75dB i.e. above this attenuation level beacons are generally not decoded successfully and so no RSSI values are reported by Bluetooth scans.

## 3 Results

### 3.1 Attenuation vs Distance

**Fig 3:**
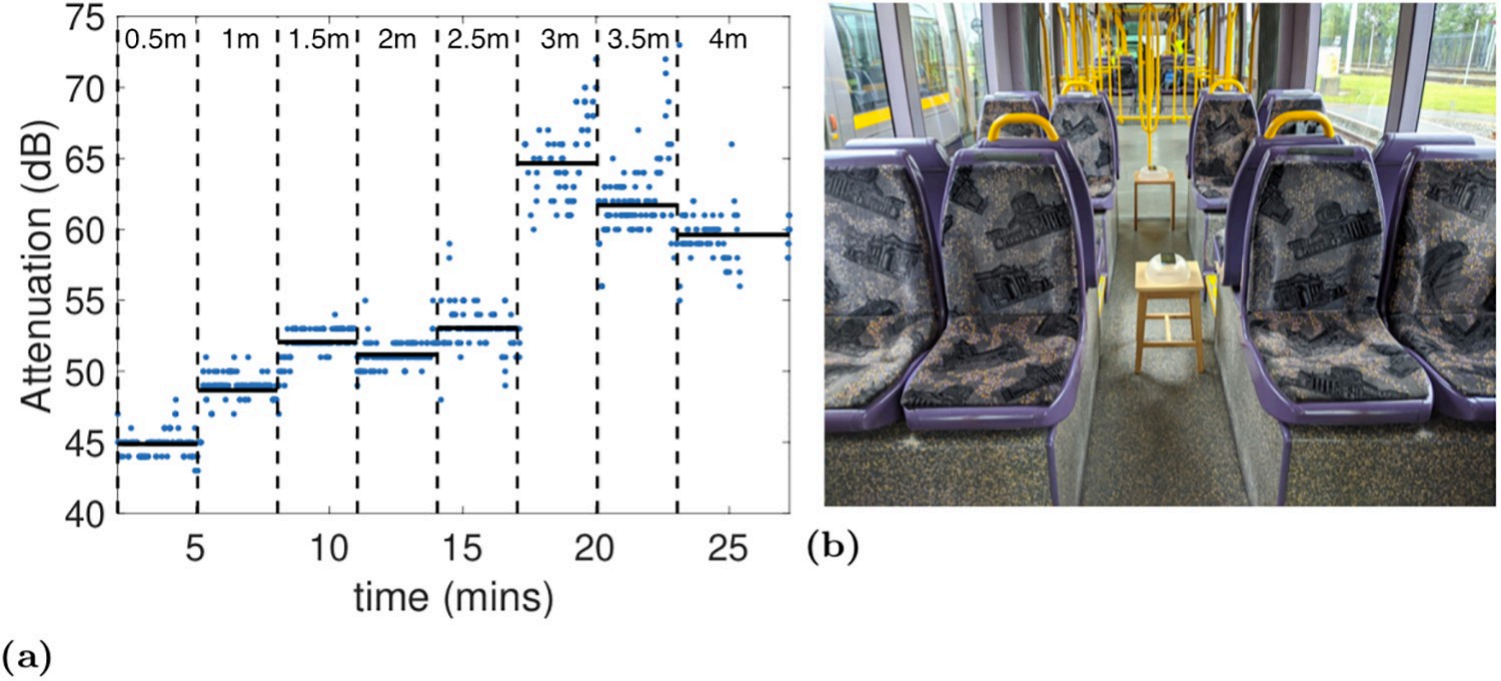
(a) Measurements of attenuation between two handsets as the distance between them is varied along the centre aisle in the tram carriage, (b) shows the setup used. The vertical dashed lines indicate when the distance between the handsets was changed, starting at 0.5m and then increasing by 0.5m at each step. The solid horizontal lines indicate the mean attenuation level at each distance. Measurements taken using the standard Android Bluetooth LE scanner API.

Fig 3(a) plots the attenuation measured between two handsets placed at seat height in the aisle of the tram as the distance between them is varied. These measurements were taken using the standard Android Bluetooth LE scanner API (rather than the GAEN API). This scanner API reports an RSSI value for each received beacon. Following [9] updated to reflect GAEN calibration changes pushed by Google on 13th June 2020, for the Google Pixel 2 handsets used in our experiments we map from RSSI to attenuation level using the formula -31-(RSSI-6) dB.

It can be seen that the attenuation initially increases as the distance is increased from 0.5m to 1.5m, as might be expected. But thereafter the attenuation level stays roughly constant with increasing distance out to 2.5m. There is then a sharp rise in the attenuation at 3m. This corresponds to the end of a group of seats and the start of a flexible joint between two carriages. As the distance is increased further it can be seen that the attenuation starts to *fall*. The attenuation is around 52dB at 1.5m and around 60dB at 4m.

The baseline measurements in Fig 3 indicate that the radio attenuation within the tram does not simply increase with the distance between handsets. This is similar to the behaviour observed in previous GAEN measurements taken on a bus [8], and is of course pertinent to the use of attenuation level as a proxy for distance. Although further measurements are needed to confirm this, it seems likely that this effect is due to reflections from the tram walls, ceiling and floor, all of which are made of metal and highly reflective at the Bluetooth radio frequency.

### 3.2 Attenuation between passengers

The full attenuation duration data reported by GAEN API is given in the Appendix and is publicly available online [5]. In this section we analyse two aspects of this data: (i) the relationship, if any, between attenuation level and distance between handsets and (ii) the magnitude of the variations in the attenuation level induced by differences in the way participants hold their handsets.

### 3.3 Trend with distance

**Fig 4:**
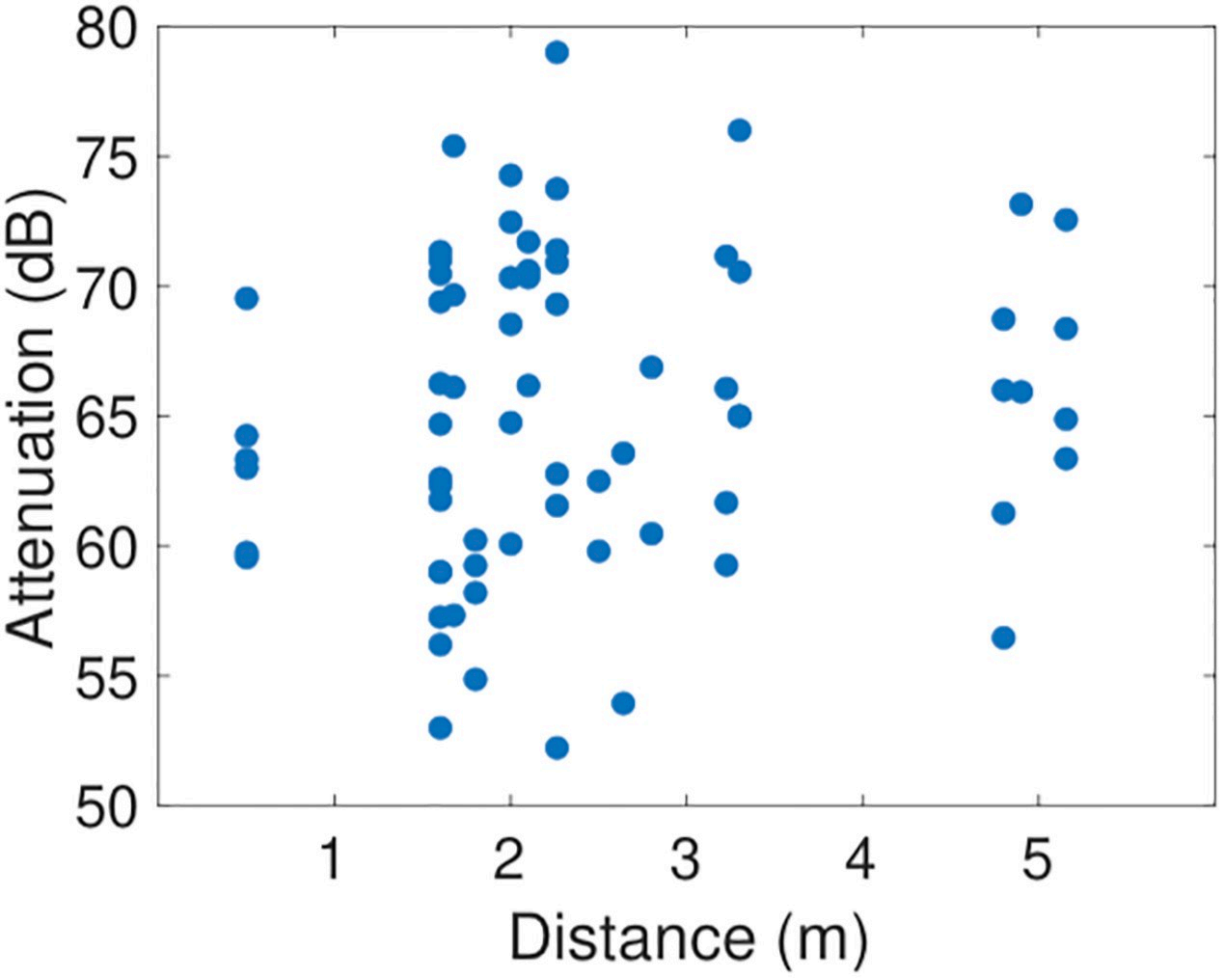
Mean attenuation level vs distance between handsets.

Fig 4 plots the mean attenuation level vs the distance between participants in the three tests. The mean is calculated by weighting each attenuation level by the duration at that level reported by the GAEN API and then summing over all attenuation levels. It can be seen that there is no clear trend in the mean attenuation level as the distance changes, with similar ranges of attenuation levels observed at all distances, except perhaps for distances below 1m where the attenuation level is more tightly clustered.

**Fig 5:**
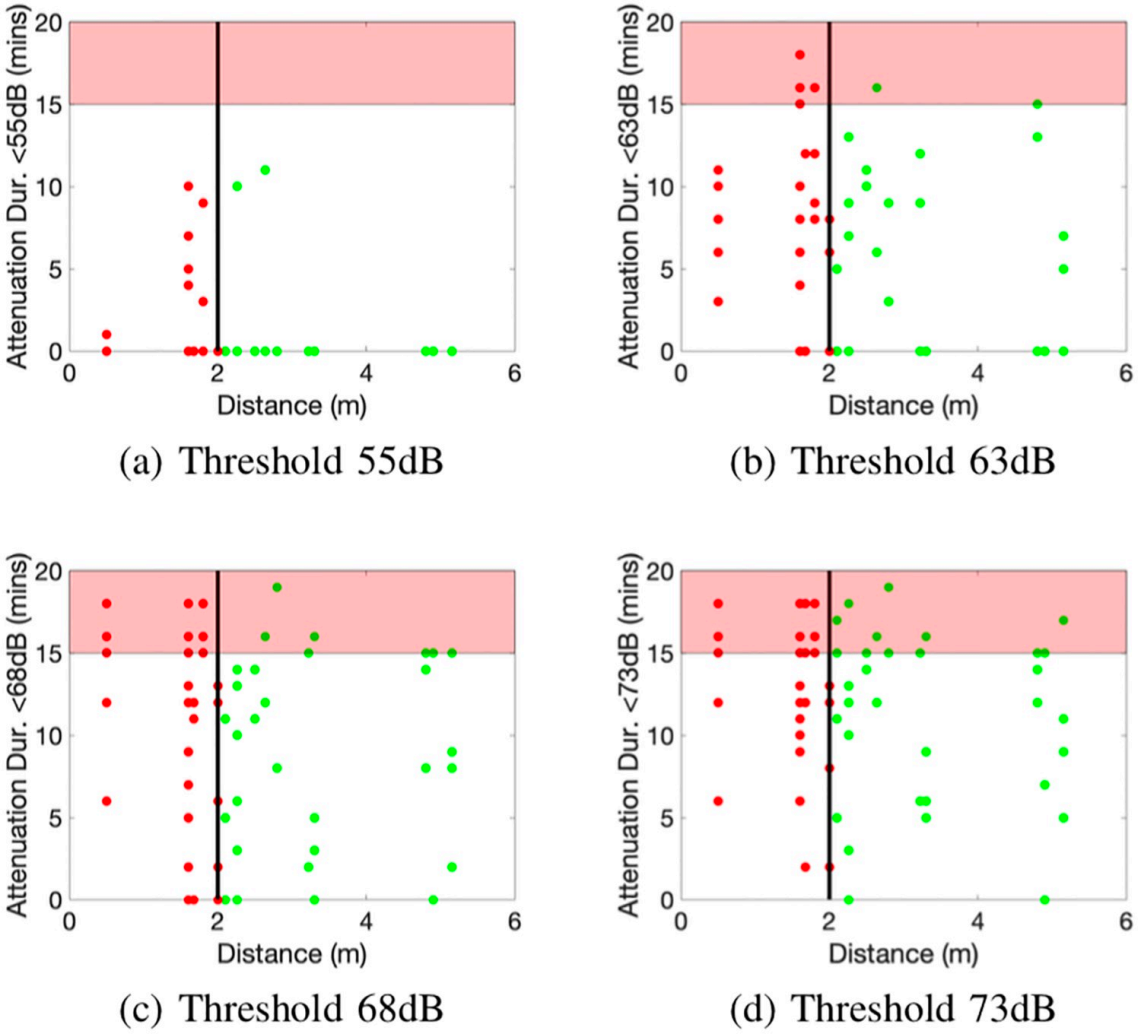
Sum-duration that the measured attenuation level is below 55dB, 63dB, 68dB and 73dB vs distance between handsets. The shading marks the area where the sum-duration exceeds 15 minutes.

The GAEN API records the duration at each attenuation, and so effectively the full distribution of attenuation levels rather than just the mean. Fig 5 plots the sum-duration that the measured attenuation level is below 55dB, 63dB, 68dB and 73dB. For each pair of handsets these values are the rescaled empirical CDF of the attenuation level evaluated at the specified values. Recall that a typical definition of a proximity event is spending 15 minutes or more at a distance of 2m or less apart. We have therefore indicated the 2m distance with a vertical line in Fig 5, and attenuation durations greater than 15 minutes by the shaded areas.

For reliable detection of proximity events what one might like is that for an appropriate choice of threshold value the attenuation levels lie within the shaded area when the distance is less than 2m and outside the shaded area when the distance is greater than 2m. Unfortunately we do not see such behaviour in Fig 5. Instead, consistent with Fig 4 we see no consistent trend between attenuation duration and distance below/above 2m.

### 3.4 Magnitude of inter-test variations

**Fig 6:**
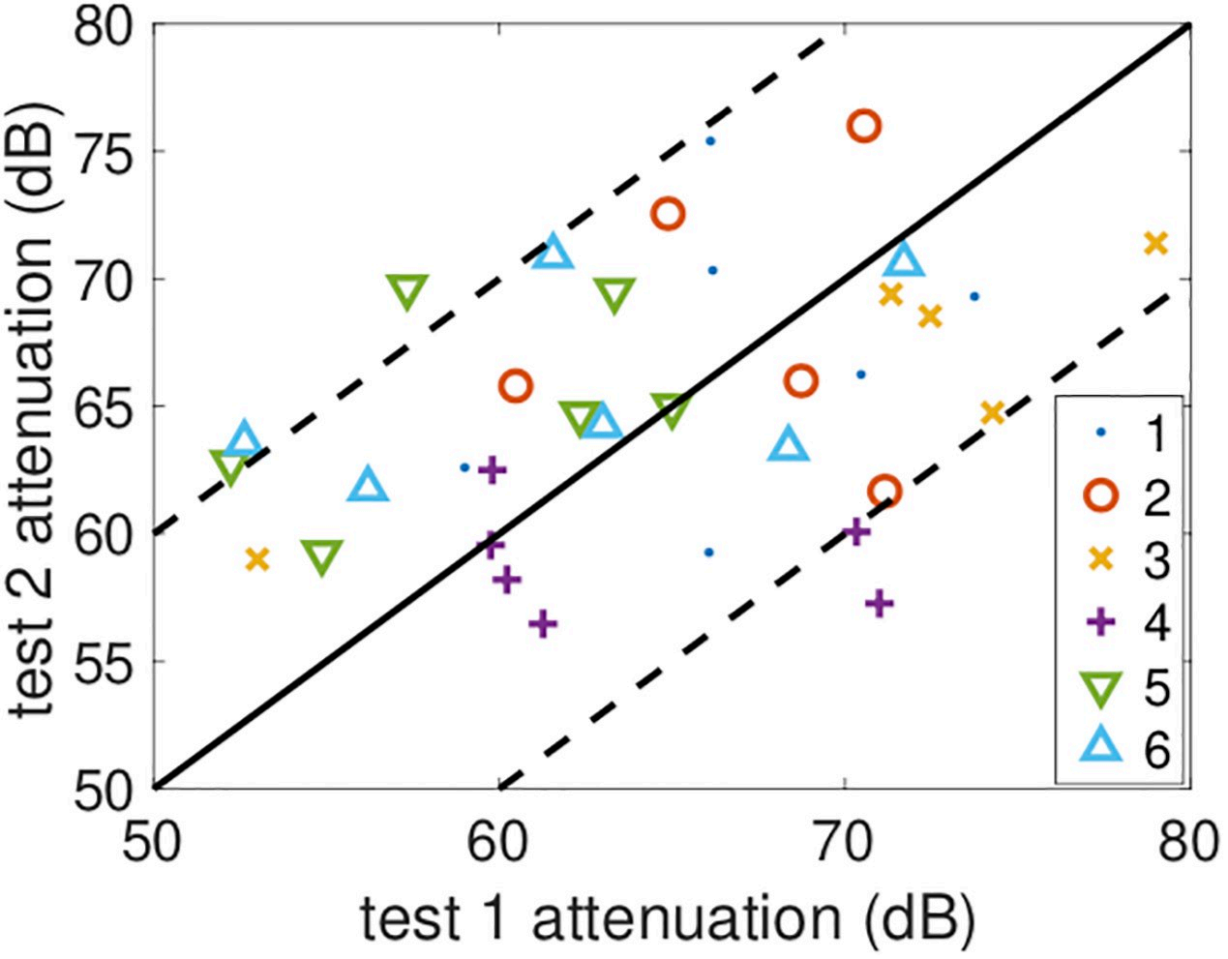
Mean attenuation level in test 2 vs test 1 for the same seat position. The legend indicates the seat index of the transmitting handset, see Fig 1(b) for the location. The solid line is the 45° line and the dashed lines the ±10dB lines about this.

Between each of the three experiments the participants switch seats. The seat positions themselves remain the same, only the person sitting in the seat changes, allowing us to see the impact of differences in the way that each participant uses their handset. For beacons transmitted from each seat position Fig 6 shows the mean attenuation level observed at the other seat positions (see the Appendix for the full attenuation duration data). The attenuation level observed in test 1 is plotted vs the attenuation level observed in test 2. It can be seen that the points are clustered around the 45° line, but variations of ±10dB between the two tests are common. Since the seating locations and environment within the tram are the same between experiments, participants have similar build and height and use the same model of handset, these variations can be attributed to differences in the way each particpant holds their handset and/or changes between tests in the way the same particpant holds their handset. Such substantial variations in attenuation level are obviously pertinent to the use of attenuation level for proximity detection.

### 3.5 Exposure notification true/false positive detection rate

The GAEN API is intended for use by health authority Covid-19 contact tracing apps [4]. When a person is found to be infected with Covid-19 the TEKs from their handset are uploaded to a central server. The health authority app on another person’s handset can then download these TEKs, and use them to compare against the set of beacons received by the handset. If there is a match, the attenuation duration values reported by the GAEN API can then be used to estimate the risk of infection and trigger an exposure notification is this risk is sufficiently high.

A typical requirement is for a person to have spent at least 15 minutes within 2m of the infected person in order to trigger an exposure notification. The mapping from GAEN attenuation durations to exposure notification is therefore largely based on use of attenuation level as a proxy for proximity between handsets.

#### 3.5.1 Swiss & German exposure notification rules

Switzerland deployed a Covid-19 contact tracing app based on the GAEN API on 26 May 2020 [15]. The documentation for this app states that it queries the GAEN API with low and high attenuation thresholds of *t*1 = 50dB and *t*2 = 55dB and then bases exposure notifications on the quantity *ES* = *B*1 + 0.5*B*2, where B1 is the attenuation duration below 50dB reported by the GAEN API and B2 is the attenuation duration between 50dB and *t*2 [16]. An exposure notification is triggered when *ES* is greater than 15 mins, see Table 1.

**Table 1:** Summary of detection rules studied. *B*1 is the the attenuation duration below threshold *t*1, *B*2 is the attenuation duration between *t*1 and *t*2, *B*3 is the attenuation duration below *t*2.

App	Detection Rule	Parameter Values
Swiss App	*B*1 + 0.5*B*2 > 15 mins	*t*1 = 50db, *t*2 = 55dB
German App	*B*1 + 0.5*B*2 > 10 mins	*t*1 = 55db, *t*2 = 63dB
Italian App	*B*3 > 15 mins	*t*2 = 73dB

Germany deployed a Covid-19 contact tracing app based on the GAEN API on 15 June 2020 [17]. The app is open source. By inspecting the documentation and code, and querying the server API to obtain the app configuration settings This means that the app configuration can be dynamically updated. We downloaded the detection settings from the server on 21 June 2020 and they are included in the study data repository [5], we determined that the German app follows an approach similar to the Swiss app for triggering an exposure notification, but uses values *t*1 = 55dB and *t*2 = 63dB and an exposure duration on 10 minutes.

**Fig 7:**
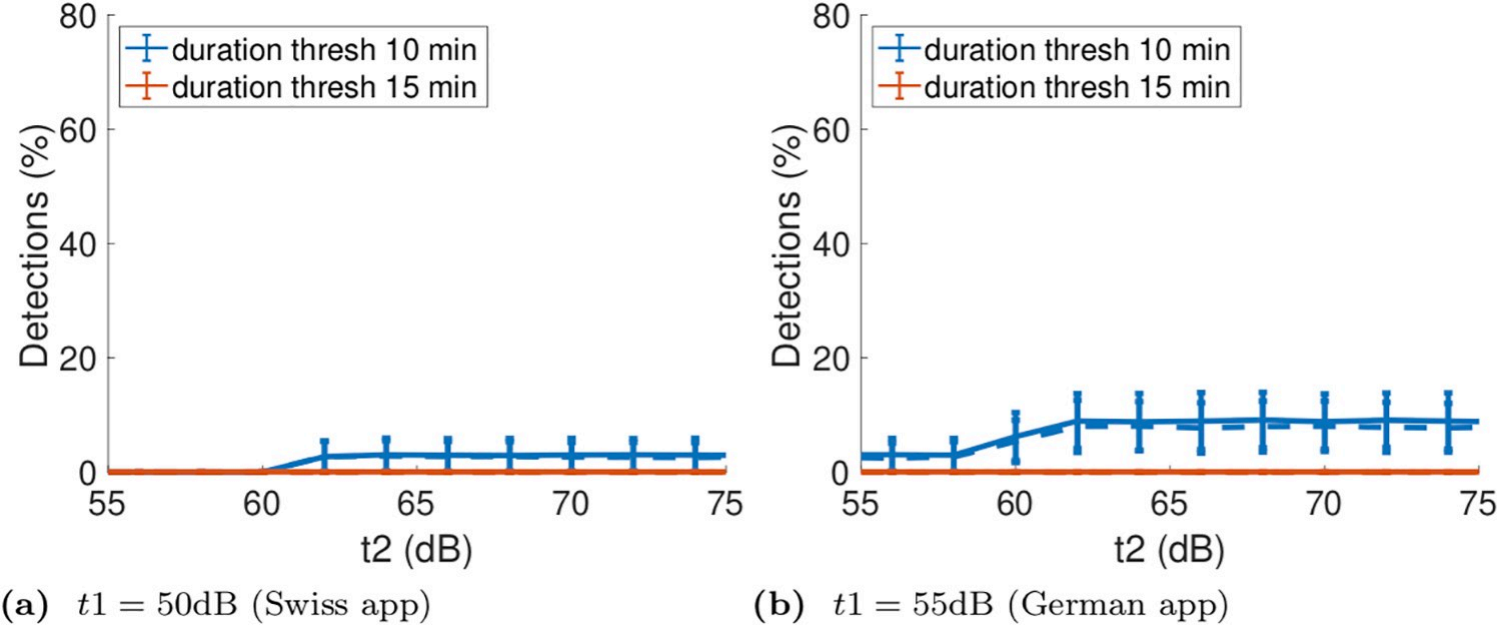
Exposure notification true and false positive rates when the threshold strategy used in the Swiss and German contact tracing apps is applied to the GAEN tram dataset. Data is shown vs attenuation level and duration thresholds, solid lines indicate true positive rates and dashed lines the corresponding false negative rates.

We applied the Swiss and German exposure notification rules to the tram dataset. Fig 7(a) plots the true and false positive rates for *t*1 = 50dB and as *t*2 is varied from 55dB upwards and the *ES* threshold varied from 10 minutes to 15 mins. The mean rates are shown with one standard deviation indicated by the error bars. The mean and standard deviation are obtained by a standard bootstrapping approach The dataset was resampled with replacement *n* = 1000 times, the exposure notification percentage calculated for each sample and then the mean and standard deviation of these *n* estimates calculated. We selected *n* by calculating the mean and standard deviation vs *n* and selecting a value large enough that these were convergent. Fig 7(b) plots the true and false positive rates when *t*1 = 55dB.

It can be seen from Fig 7(a) that selecting *t*1 = 50dB and *t*2 = 55dB (the values used in the Swiss app) yields no positive detections, despite approximately 50% of the handset pairs in the tram dataset being within a 2m distance of one another. Increasing *t*2 to 62*dB* and above yields a small increase in detection rate, with true and false detection rates roughly equal (we comment further on the implications of this below).

It can be seen from Fig 7(b) that selecting *t*1 = 55dB and *t*2 = 63dB (the values used in the German app) there are are no detections when the threshold for *ES* is 15 minutes but when a threshold of 10 minutes is used, as in the app, then the true and false positive detection rates both rise to 9%. Increasing *t*2 does not increase these detection rates.

#### 3.5.2 Italian exposure notification rule

Italy deployed a Covid-19 contact tracing app based on the GAEN API on 1 June 2020 [18, 19]. The app is open source. By inspecting the documentation and code, and querying the server API to obtain the app configuration settings We downloaded the detection settings from the Italian app server on 21 June 2020 and they are included in the study data repository [5], we determined that the app follows a different approach to the Swiss and German apps, triggering an exposure notification whenever the attenuation duration is above threshold *t*2 = 73dB i.e. without the weighting of 0.5 used in the Swiss and German exposure notification rules.

**Fig 8:**
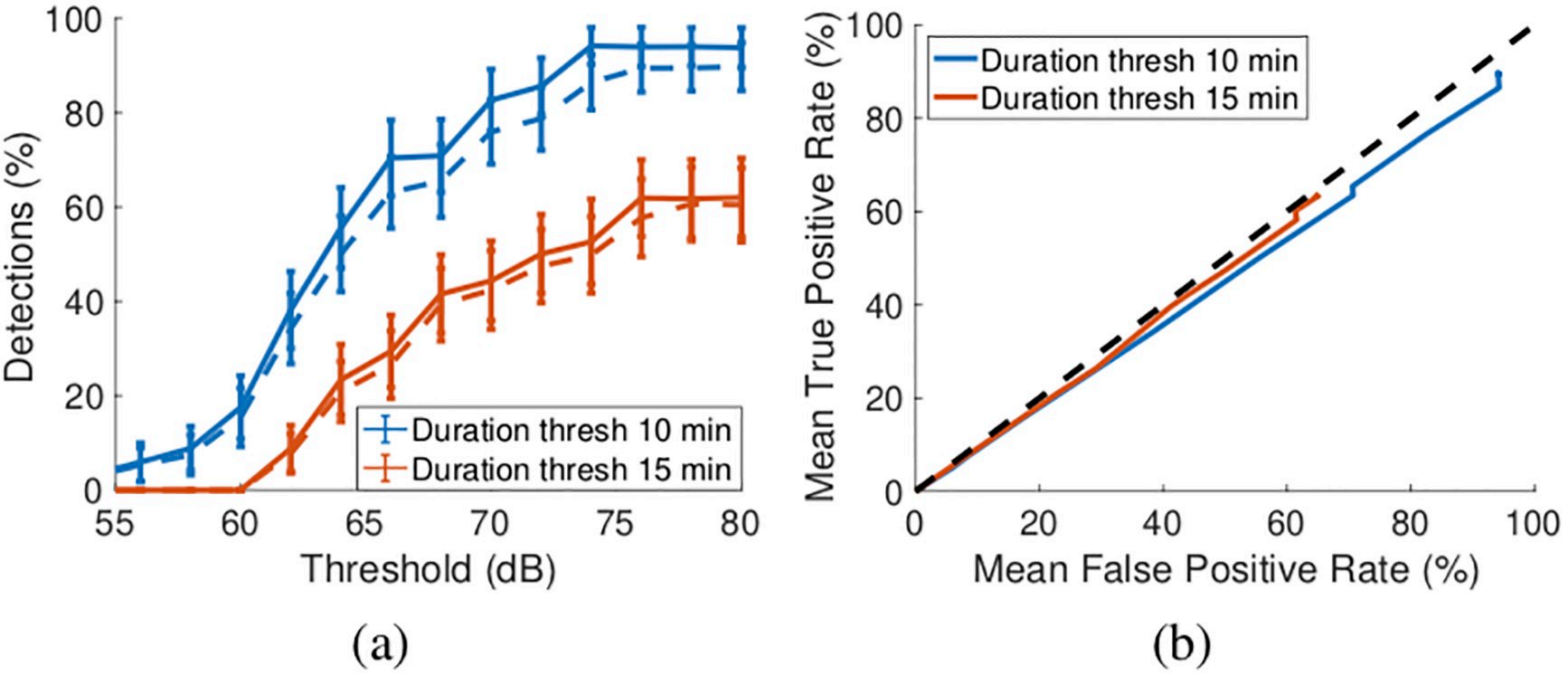
Exposure notification true and false positive rates when a simple threshold strategy is applied to the GAEN tram dataset. (a) True and false positive rates vs attenuation level and duration thresholds, solid lines indicate true positive rates and dashed lines the corresponding false negative rates (the Immuni app uses a 73dB threshold). (b) ROC plot corresponding to mean rates in (a), dashed line indicates 45° line.

We applied this exposure notification rule to the tram dataset. Fig 8(a) plots the true and false positive rates as threshold *t*2 is varied from 55dB upwards and the threshold for *ES* is varied from 10 minutes to 15 mins. For *t*2 = 73dB the true and false positive detection rates are both around 50% when the threshold for *ES* is 15 minutes, rising to 80% when the threshold for *ES* is reduced to 10 minutes. As noted in Section 2.5, with the calibration values used in the GAEN API the maximum observable attenuation level with Google Pixel 2 handsets is around 75dB (above this level beacons are generally no longer successfully received). Selecting *t*2 = 73dB therefore means that almost the full range of possible attenuation levels will trigger an exposure notfication. High detection rates are therefore unsurprising, but the detection has little discrimination and essentially would trigger exposure notifications for all participants in our tests regardless of proximity.

Fig 8(a) also shows the true and false positive detection rates for other choices of threshold *t*2. While the detection rates are generally substantially higher than with the Swiss and German detection rules, it can be seen that the false positive rate increases almost exactly in line with the true positive rate. This can be seen more clearly when this data is replotted in ROC format, see Fig 8(b). It can be seen that the true vs false positive curve lies close to the 45° line (to avoid clutter we do not plot the error bars from Fig 8(a) on Fig 8(b) but the small deviation from the 45° line is not statistically significant.). That is, the detection performance is poor, and comparable to simply selecting from participants at random when making exposure notifications.

## 4 Discussion

A limitation of this study is that it is confined to handsets using the Android operating system. The GAEN API is also implemented on Apple iOS devices, but Apple have severely limited the ability of testers to make measurements (each handset is limited to querying the GAEN API a maximum of 15 times a day, and Apple has no whitelisting process to relax this constraint. Our measurement approach uses 34 queries to extract fine-grained attenuation data per pair of handset locations.

We equipped participants with the same model of handset in order to remove this as a source of variability in the data and instead focus on variability caused by the radio environment and the way that people hold their handsets. Google and Apple are currently undertaking a measurement campaign to select calibration values within the GAEN API with the aim of compensating for differences between handset models. We therefore expect that our measurements should also be applicable to a range of handsets, although this remains to be confirmed.

With regard to calibration, we note that Bluetooth received signal strength is affected by several factors including (i) differences between different models/makes of handset, (ii) fluctuations in the relative orientation of handsets (even small changes can have a large impact), (iii) absorption by human bodies (especially when phone is in a pocket), bags etc, (iv) radio wave reflection from walls, floors, furniture. See [14] for measurements highlighing the potential for significant impact of these factors. Calibration may mitigate (i), although this remains unclear at present and variations between handsets might be expected to degrade performance compared to our measurements, but not (ii)-(iv).

In both the tram measurements reported here and previous measurements in a commuter bus [8] only a weak correlation between received signal strength and distance between handsets is observed. A direct comparison of detection accuracy in these two datasets is unfortunately not possible since in the bus measurements all pairs of handsets were within 2m of one another and so only the rate of false negatives can be evaluated.

## 5 Conclusion

We report on the results of a Covid-19 contact tracing measurement study carried out on a commuter tram in Dublin, Ireland. Our measurements indicate that in the tram there is little correlation between received signal strength and distance between handsets. We applied the detection rules used by the Italian, Swiss and German apps to our measurement data and also characterised the impact on performance of changes in the parameters used in these detection rules. We find that the Swiss and German detection rules trigger no exposure notifications on our data, while the Italian detection rule generates a true positive rate of 50% and a false positive rate of 50%. Our analysis indicates that the performance of such detection rules is similar to that of triggering notifications by randomly selecting from the participants in our experiments, regardless of proximity.

## Appendix

### Data presentation format

We present the full attenuation duration data using a coloured heatmap. We split the range of attenuation values into 2dB bins, i.e. 70-72dB, 72-74dB and so on, up to 80dB when 5dB bins are thereafter used since the data is noisier at these low signal levels. Within each bin the colour indicates the percentage of the total duration reported by the GAEN API that was spent in that bin, e.g bright green indicates that more than 90% of the time was spent in that bin. The mapping from colours to percentages is shown on the righthand side of the plot. Bins with no entries (i.e. with duration zero) are left blank. Where appropriate we also include a solid line in plots that indicates the average attenuation level at each transmit power level (the average is calculated by weighting each attenuation level by the duration at that level and then summing over all attenuation levels).

**Fig 9:**
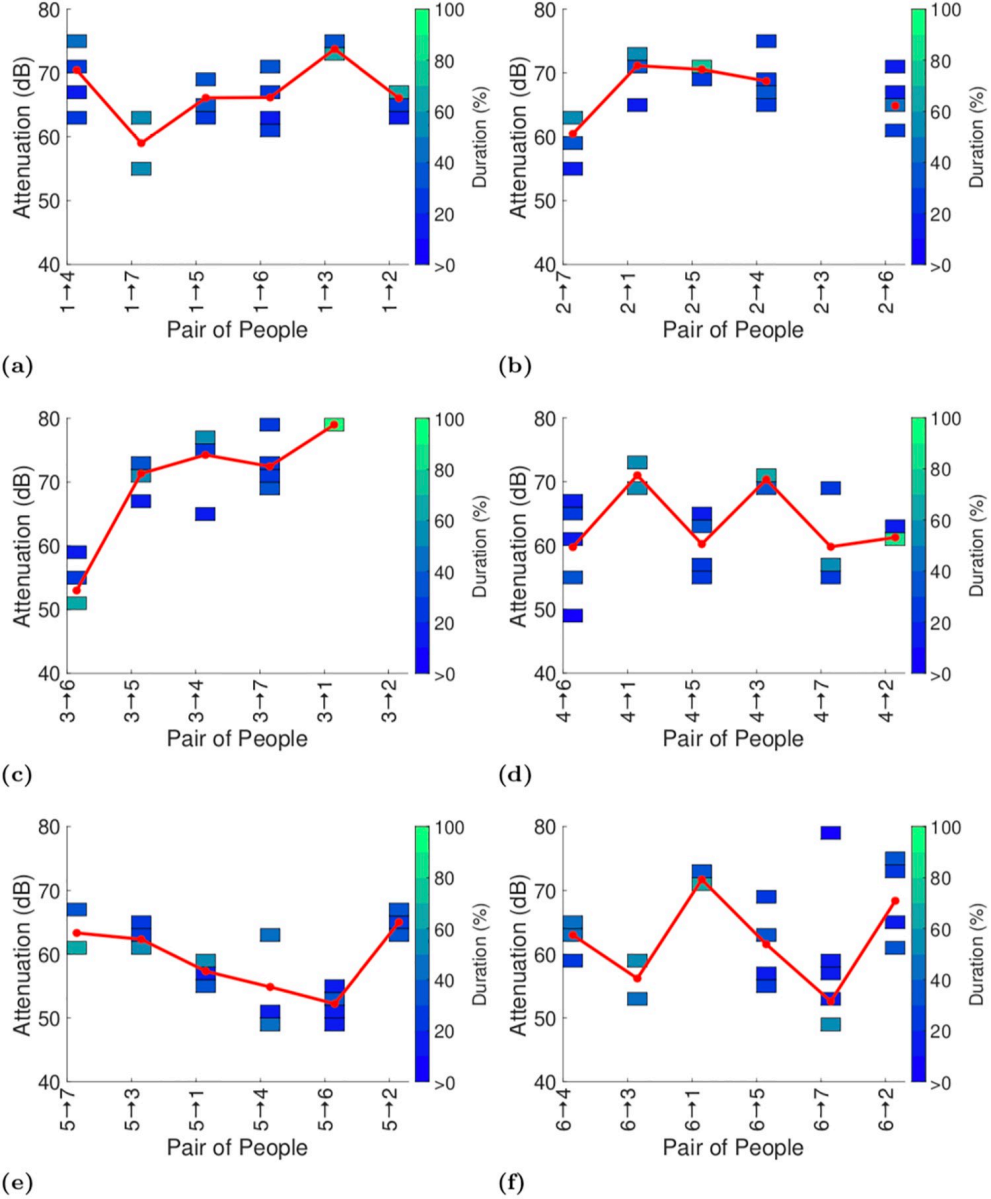
Attenuation durations reported by GAEN API on completion of the first test. Pairs indicated on x-axis in each plot are ordered by increasing distance.

For example, in Fig 9(a) the left-hand heatmap shows the attenuation durations measured between participants 1 and 4. The attenuation spends around 60% of its time in the 74-76dB bin, with the rest of the time roughly evenly split between 62-64dB, 66-68dB, 70-72dB. The weighted average attenuation is 70dB.

### Measurement data

**Fig 10:**
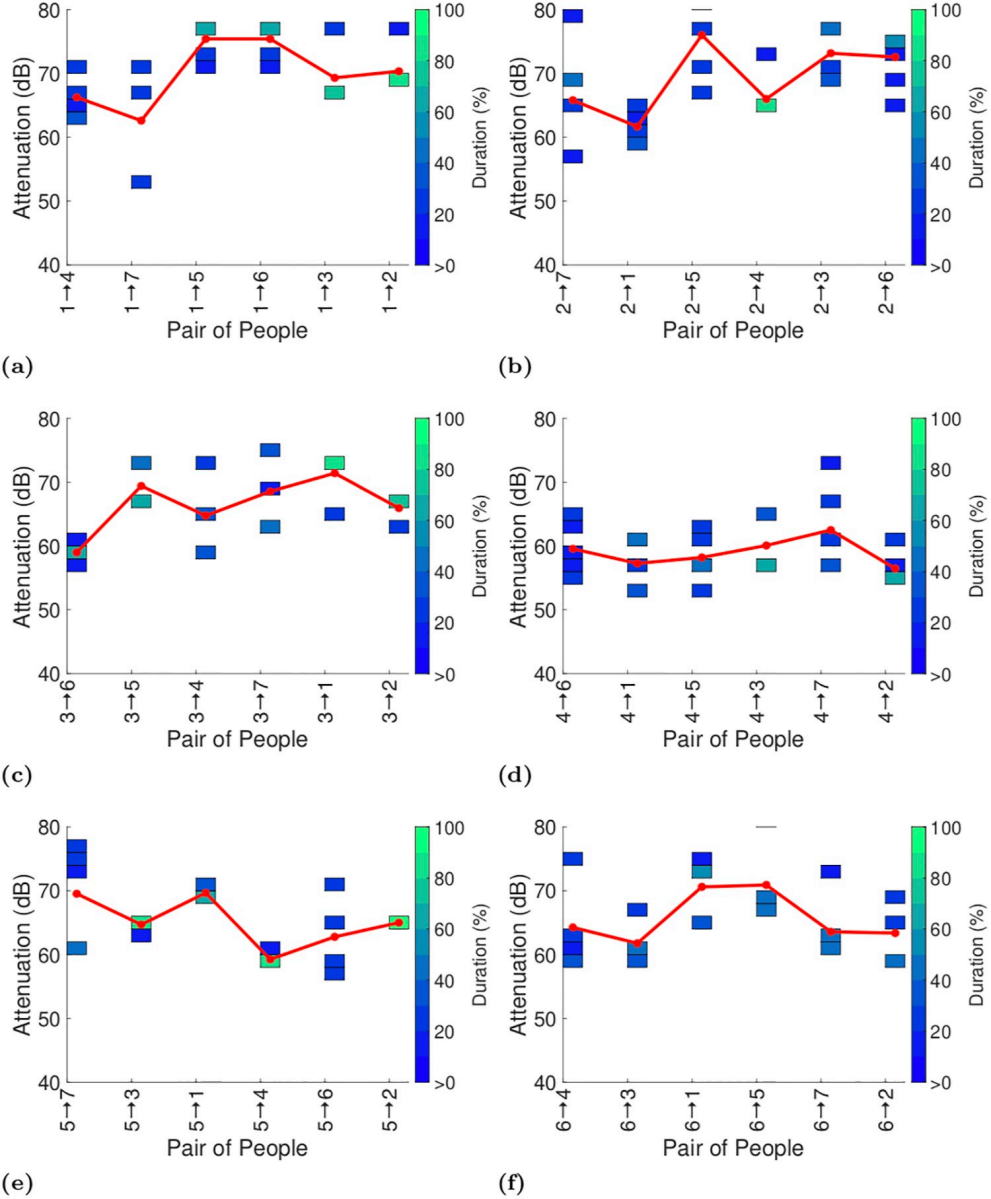
Attenuation durations reported by GAEN API on completion of the second test (with the same participants as in the first test, but with their seating positions swapped about). Pairs indicated on x-axis in each plot are ordered by increasing distance.

**Fig 11:**
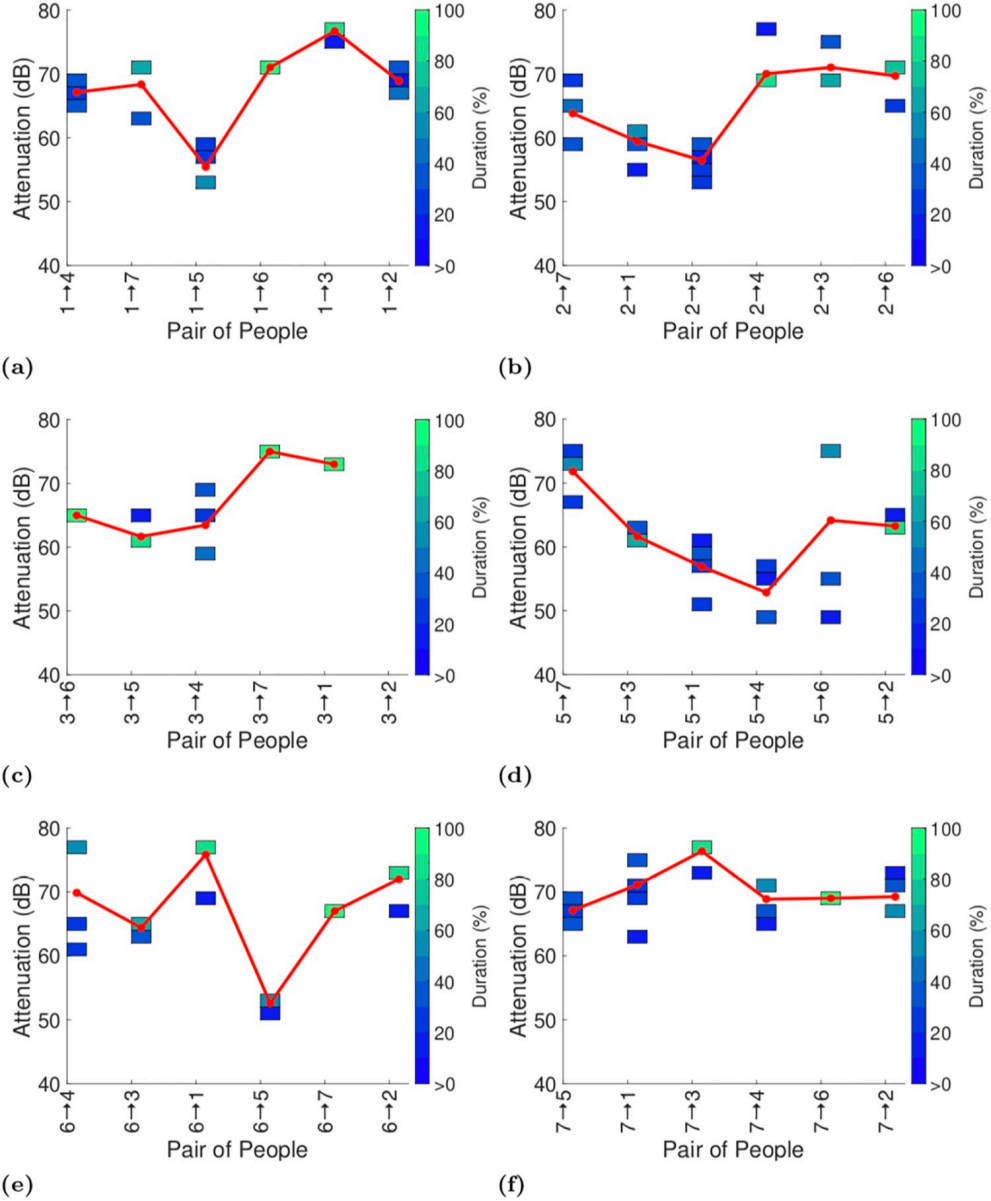
Attenuation durations reported by GAEN API on completion of the third test (with the same participants as in the first test, but with their seating positions swapped about and participants 1 and 3 with handsets in their trouser pocket rather than their hand). Pairs indicated on x-axis in each plot are ordered by increasing distance.

Figs 9–11 plot the exposure information between each pair of handsets reported by the GAEN API for each of the three experiments. To assist with interpreting the plots the reports in each plot are ordered by increasing distance between the pairs of participants (see Fig 1(a)). No data is shown when no beacons were received between a pair of handsets, e.g. between particpants 2 and 3 in Fig 9(b) and 9(c). It can be seen that occasionally there is an increasing trend in attenuation, for example see Figs 9(c) and 11(c), but this is infrequent. Occasionally there is a decreasing trend in attenuation, for example see Figs 9(e) and 11(d). Overall, however no consistent trend is evident in the change in attenuation level with increasing distance.

In Fig 11 participants 1 and 3 place their handsets in their left trouser pocket rather than their hand. Intuitively, one might expect this change to increase the attenuation level since the particpants body is now more likely to affect transmission and reception of radio signals. However, comparing Fig 11(a) and 11(c) with Figs 9 and 10 it can be seen that this change does not cause any consistent change in the observed attenuation level. For example, comparing Figs 11(a) and 10(a) the attenuation level between participants 1 and 5 decreases from test 2 to test 3, while the attenuation level between participants 1 and 3 increases.
